# Obituary

**Published:** 1852-06

**Authors:** 


*Obituary.*

Died on the evening of the 2d of May last, at the residence of his father in Bruceville, Knox Co., Ia., Mr. James L. Thompson, in the 21st year of his age.

Mr. T. had made choice of the profession of medicine, and having completed his preparatory collegiate course, had pursued the study term two years, under the tutorage of his father, Dr. T. B. Thompson, and had attended the winter course of lectures of '51-2, in the Rush Medical College of Chicago, Ill. Having formed a partiality for the professors in that institution, his calculation was to have graduated there in the spring of '53. But an all wise and overruling Providence had decreed otherwise, and he was summoned to attend at a higher tribunal.

Mr. T. was a young man of much promise, of untiring industry and perseverance; partial to the profession of his choice, he was indefatigable in his exertions to become master of its intricacy; with a mind above the ordinary grade, of remarkably steady habits, and a moral character unexceptionable, his prospects for future usefulness were more than ordinarily bright; in short, he bid fair to become a useful and an ornamental member of the profession.

His disease, which was pneumonia, of fourteen days' continuance, terminating in effusion of the lungs, was borne with Christian fortitude and resignation. *

Bruceville, May 20, 1852.

